# Penetrating Injury of Pelvis, Abdomen and Thorax in a Child with a Trident (Trishula)

**Published:** 2013-01-01

**Authors:** Jile Dar Rawat, Prabudh Goel, Vijaymahantesh S. Kunnur, BB Kushwaha, Renu Kushwaha

**Affiliations:** Dept. of Pediatric Surgery, King George’s Medical University (Upgraded King George’s Medical College), Lucknow. India; Dept. of Pediatric Surgery, King George’s Medical University (Upgraded King George’s Medical College), Lucknow. India; Dept. of Pediatric Surgery, King George’s Medical University (Upgraded King George’s Medical College), Lucknow. India; Dept. of Anesthesia, King George’s Medical University (Upgraded King George’s Medical College), Lucknow. India; Dept. of Pediatric Surgery, King George’s Medical University (Upgraded King George’s Medical College), Lucknow. India

**Keywords:** Thoraco-abdomino-pelvic impalement, Child, Injury, Trident, Trishula

## Abstract

Penetrating abdominal injuries are uncommon in the pediatric age group and are associated with a high mortality. A seven year old girl suffered penetrating injury to perineum when she fell onto the trident (trishula – traditional three pronged spear) implanted alongside Lord Shiva’s idol. The rod caused perforations of the transverse mesocolon, ileum at 3 places, right lobe of liver, diaphragm, parietal pleura, and exited from 7th intercostal space. Surgical repair of each damaged site was undertaken. Despite delayed presentation, the child survived following surgery.

## INTRODUCTION

Impalement injuries are extremely rare in the pediatric age-group and are associated with high mortality [1]. We share our experience in a seven year old girl who suffered perineal impalement traversing the pelvic, abdominal, and thoracic cavities in a remote village.

## CASE REPORT

A 7-year-old girl from a remote village presented almost 16 hours after an impalement injury. The child was enjoying the swing by hanging from the shoots of the banyan tree. The shoot got disconnected from the tree and the child fell onto the trishula implanted alongside Lord Shiva’s idol under the Banyan tree (Fig. 1). The trishula entered the pelvis lateral to the midline between the anal orifice and the right ischial tuberosity in the right gluteal region, traversed the abdomen and made an exit through the 7th intercostal space at right sided anterior axillary line. The worshippers in the temple fortunately, decided to dismantle the ‘trishul’ from the ground while it remained in situ. In the absence of any medical facilities locally, the parents rushed the child to our centre which was about 500 kms from the site of event and took about 16 hours by local transport. The child presented on a wooden board with the trishula in-situ. She was lying in lateral cuddled up position and was in severe pain though hemodynamically stable (Fig. 1). She was pale and tachypneic. The entry and exit wounds were oozing fresh and clotted blood. The entry wound was about 3cm in diameter. The exit wound was 1.5cm in diameter. Both the wound openings were sealed by the iron rod. The abdomen was not distended. 

**Figure:**
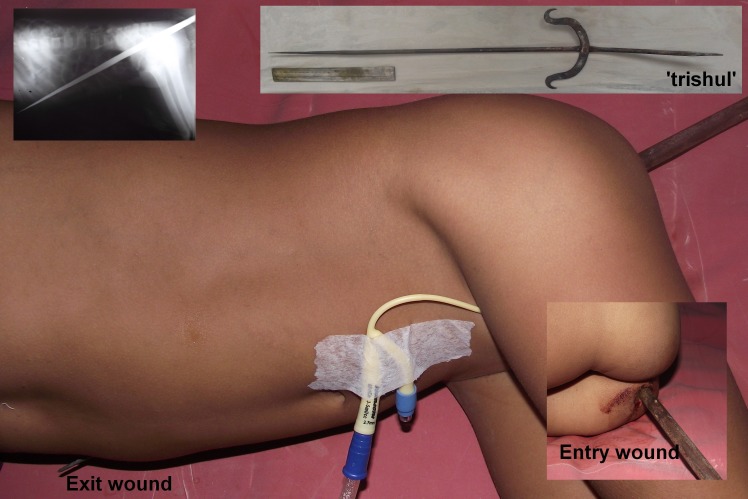
Figure 1: Pre-operative picture of the patient. Inset pictures show the trishula, body radiograph, and the entry and exit wounds.

Patient was given IV fluids and blood transfusions. Portable abdominal and chest skiagrams were obtained which did not show any bony injury, hemothorax, pneumothorax, or pneumoperitoneum. Tetanus toxoid was administered and the child underwent exploratory surgery through midline. The rod had entered into the abdomen just lateral to the rectum sparing its wall and perforating the peritoneal reflection. The transverse mesocolon was perforated close to the bowel wall resulting in serosal injuries on the mesenteric border of the transverse colon but the mucosal continuity was not interrupted. Thereafter, the small bowel was perforated at three sites (45, 18cm and 9cm proximal to the ileo-caecal junction). The perforations were sealed by the iron rod preventing any peritoneal contamination. Subsequently, the rod traversed through segments 6 and 7 of the liver entering from the inferior surface and exiting from the antero-superior surface. The diaphragm and the parietal pleura were perforated but the lungs were spared. The rod exited from the 7th intercostal space in the right anterior axillary line (Fig. 2). 

**Figure:**
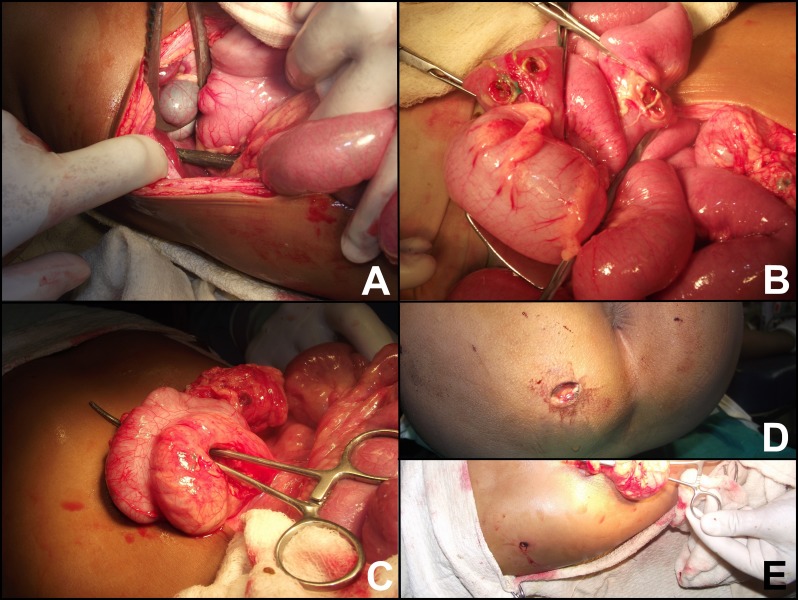
Figure 2: (A) The trishula is seen perforating the right lobe of the liver, (B) The small bowel perforated at three different places, (C) Perforated transverse mesocolon, (D) Entry wound, (E) Exit wound located at the right 7th intercostal space.

The iron rod was gradually withdrawn under direct observation. Pleural drain was inserted and connected to under-water seal. The diaphragmatic rent was repaired with polyglycolic sutures (Vicryl - Ethicon). Withdrawal of the rod from the right lobe of the liver resulted in hemorrhage which was managed by packing with multiple gauzes for a couple of minutes to ensure hemostasis and two surgicel (tightly woven knitted patch of oxidized cellulose with hemostatic and anti-bacterial properties) were left in-situ. The rents in the bowel wall were freshened and repaired in two layers. The mesenteric defect was repaired. The entry and exit wounds were irrigated with hydrogen peroxide and packed with betadine soaked gauzes. Pelvic and right paracolic tube-drains were secured and the abdomen closed after thorough peritoneal lavage. 

The postoperative recovery was rapid and uneventful. Oral diet was initiated on the fifth postoperative day. Drains were removed after feeding was established. The patient was discharged on 8th postoperative day. Three months after the accident, the patient is thriving well.

## DISCUSSION

Perineal impalement injuries with penetration of the thoraco-abdomino-pelvic regions are extremely uncommon. Impalement injuries are consequence of penetration of elongated, usually fixed objects through the body. Firearm, projectile missile injuries are excluded from the definition of impalement injuries. Stab wounds with the weapon in-situ, however are included in the spectrum of impalement injuries [1]. An impact between the human body and the object such as seen in cases of road traffic accidents or accidental fall from height is the usually encountered mechanism. Similar accident occurred in the index case.

Pre-hospital care is very crucial to the survival of these patients. There are certain basic principles which must be adhered to in such a situation. Care must be taken not to remove the penetrating object while patient is being transported to nearby hospital where definite treatment is to be provided otherwise fatal hemorrhage may occur [2]. The impaling object may cause a tamponade effect on the organs through which it has penetrated, thus preventing bleeding following trauma. As a rule, the impaling object must be removed under direct vision in a controlled environment such as in the operation theatre. Moreover, with the foreign body in place it is always easier to physically visualize the organs through which it has traversed, thus preventing missing of any organ injury during surgery. There are very few exceptional situations wherein immediate removal of the impaling object is indicated such as if the patient needs cardio-pulmonary resuscitation and the object is interfering with it or if the impaling object is in the way of the patient’s airway. Shortening of the object may however be attempted to facilitate transport. The impaling object must be secured so as to prevent any movement in relation to the body of the patient. This is to prevent further soft-tissue damage and bleeding. In the index case, the relatives did not make any attempt to remove the impaling object thus helped in limiting further injuries. 

Impalement abdominal injuries are acute emergencies. The basic principles of trauma care apply to the management of these injuries. Assessment of the hemodynamic status as well as the amount of blood lost directs the initial management and stabilization of the patient while preparations for the surgery are being done. Administration of tetanus toxoid and broad spectrum antibiotic coverage is justified by the highly infective potential of the impaling objects. The operative approach is determined on the basis of the tract of the impaling object [1, 2]. We chose a midline laparotomy which can be extended in either direction depending upon the need. The trishula in our case sealed all the bowel rents preventing escape of fecal material into the peritoneal cavity. We could repair all bowel injuries primarily without any consequence. Fortunately, the trishul had not disrupted any major blood vessel, nerves or spine. Liver was the only solid organ injured. At laparotomy, the presence of the trishula facilitated the identification of all the injuries that were repaired successfully.

## Footnotes

**Source of Support:** Nil

**Conflict of Interest:** None declared
